# The workforce trends of nurses in Lebanon (2009–2014): A registration database analysis

**DOI:** 10.1371/journal.pone.0182312

**Published:** 2017-08-11

**Authors:** Mohamad Alameddine, Nariman Chamoun, Rachel Btaiche, Nour El Arnaout, Nathalie Richa, Helen Samaha-Nuwayhid

**Affiliations:** 1 Department of Health Management and Policy, Faculty of Health Sciences, American University of Beirut, Beirut, Lebanon; 2 College of Medicine, Mohammed Bin Rashid University of Medicine and Health Sciences, Dubai, United Arab Emirates; 3 Calcium Metabolism and Osteoporosis Program, American University of Beirut, Beirut, Lebanon; 4 Order of Nurses in Lebanon, Beirut, Lebanon; Public Library of Science, FRANCE

## Abstract

**Background:**

Analysis of the nursing registration databases is a highly informative approach that provides accurate and reliable information supporting evidence based decisions relevant to the nursing workforce planning, management and development. This study presents the first systematic analysis of the nursing registration database in Lebanon. It Reports on the workforce distribution and trends using an updated version of the Order of Nurses in Lebanon (ONL) databases.

**Methods:**

This study presents a secondary data analysis of a de-identified subset of the updated ONL registration database. The workforce participation status of ONL registered nurses was categorized as active and eligible. For active nurses sectors and sub-sectors of employment were defined. Eligible nurses were categorized as unemployed, working outside nursing and working abroad. SPSS was used to conduct descriptive analysis to present workforce trends of Lebanese nurses for year 2009–2014 as frequencies, percentages and percentage changes.

**Results:**

Increases in the size of the Active (35%) and Eligible (86%) nurses were observed over the past six years. The majority of nurses fell in the below 35 years age group (60% in 2014). The hospital sector remained the principle employer, with 87% of Lebanese nurses working in hospitals in 2014. A 173% increases was reported for nurses working abroad.

**Discussion:**

Despite the growth of the Active nursing workforce, the skewed distribution of nurses in the below 35 age group and the growth in the Eligible category, especially for nurses living abroad, raise concerns on the longevity of nurses in the profession and the reasons for their attrition from the workforce.

**Conclusion:**

There is a need to investigate the push and pull factors that are affecting nurses and the design of policies and interventions that would encourage nurses to remain active in Lebanon. Furthermore, policies and interventions that would create employment opportunities outside hospitals, especially in the Community sector, are recommended.

## Introduction

Nurses play a leading role in health interventions and assist in the improvement of health outcomes for individuals, families, and communities [1–3]. The adequate supply of nurses is pivotal to strengthening health systems towards improved health coverage and achievement of targets. One highly informative approach to support evidence-based decisions relevant to the nursing workforce is the analysis of nursing registration databases. The advantage of such an analysis is that it delivers accurate and reliable data supporting the generation of systematic recommendations, the forecasting of future needs and the formulation of policy and practice recommendations that would slow the attrition of nurses and minimize nursing shortages [4, 5]. Data from nursing registration databases is gathered, analyzed and utilized to improve the nursing workforce planning and the quality of nursing care [6, 7].

Studies that resorted to the analysis of nursing registration databases enabled the identification of important trends in the nursing workforce, including: the ageing of the workforce, early retirement trends and gender trends [8–10]. In addition to the workforce “greying” issues, much attention has been dedicated to analyzing the shift in the nursing workforce from one care sector to another, especially from hospitals to the community setting [11]. Analysis of nursing registration databases also enabled estimating the proportion of inactive nurses (those registered and not working as nurses in the labor market) and the required strategies that would support their workforce reintegration [12].

### Lebanese nursing labor market

The nursing labor market in Lebanon suffers a number of challenges including poor supply, shortages and brain drain. For instance, the ratio of nurses to population in Lebanon is 2.72 nurses to every 1000 population, which is relatively low compared to 8.02 and 4.49 nurses to every 1000 population in Europe and North America; respectively [13]. This is disconcerting taking into consideration the steady increase in the population, the constant influx of refugees into the country, a surge in communicable and non-communicable diseases and the ageing of the population [2, 14]. Adding to the shortage issue, the Lebanese nursing workforce suffers from low enrollment in the nursing program, high migration, and low retention rates [15, 16]. Literature reports that 20% of Lebanese nurses graduating with a Bachelor’s degree migrated from the country within one or two years after graduation [15, 17]. Studies have also suggested that significant increases in the nursing workforce density is needed to fill in the need gap in Lebanon [18]. The creation of an updated national database of Lebanese nurses is flagged as a prerequisite to facilitate the evidence-based planning of the nursing workforce [15, 17, 19].

### The Order of Nurses in Lebanon

The Order of Nurses in Lebanon (ONL) is the regulatory body of nurses. It was established in 2002 with the mandate of governing and distinguishing the nursing profession, setting the professional standards and encouraging the retention of nurses within their working environment. The registration of nurses with the ONL is a pre-requisite for practicing in Lebanon. To register with ONL, each nurse fills a registration form, pays the annual subscription fee and is subsequently assigned a unique registration number. According to the Lebanese Decree 1655 on the Regulation of the Nursing, the ONL grants the title of “Registered Nurse (RN)” if one of the following two conditions is fulfilled:

1. The applicant is a holder of the Lebanese baccalaureate certificate or its official equivalent upon completion of 12 years of school education, and obtained afterwards a Bachelor’s in Nursing (BSN) degree upon completion of the requirements of a 3-year nursing program in a university acknowledged by the Lebanese government and the applicant have passed the licensure government examination [20].
2. The applicant is a holder of high diploma in nursing issued from the Ministry of Vocational and Technical Education upon completion of the requirements of a 3-year Technical Baccalaureate Diploma in Nursing (BT) after 9 years of school education, followed by 3-year Superior Technician Diploma (TS) in Nursing, and the applicant have passed the licensure government examination. Both BT and TS are obtained upon enrollment in technical programs at centers for technical sciences [20].

### Objective

This study is the first to examine the trends and distribution of the nursing workforce in Lebanon, through an in-depth secondary data analysis of six-year linked data retrieved from the ONL registration databases. It presents an overview of the trends on the supply, demographics, distribution, and education of Lebanese nurses.

## Methodology

### The ONL registration database

The ONL maintains a database for all nurses registered to practice in Lebanon that includes some information about their demographics, education and work/employment. Given that it was crucial to ensure that the data included in the analysis of this study is up-to-date, valid, and reliable, the ONL carried out, a comprehensive cleaning and updating process for the records of its registration database prior to conducting this study as an initiative to provide this project with most representative data possible. The cleaning and updating process of the registration records conducted by the ONL aimed at minimizing the number of outdated data and undisclosed or unknown registrants, and entailed ONL hired operators contacting nurses registered with the ONL by phone to update their registration information. Four operators were hired and trained by the ONL to call the registered nurses, update their records and understand their employment conditions and all phone contacts were carried out at the ONL premises. The ONL database cleanup protocol entailed categorizing nurses as “unknown” after five unsuccessful attempts to reach them by phone. The ONL database cleaning project was performed internally by ONL as a performance improvement initiative approved by the ONL Council. The research team only assumed an advisory role to the council during the cleanup phase with no access to the databases and no involvement in the actual database cleanup work.

### Ethical considerations

Employing a secondary data analysis study design, our study was deemed exempt from the Institutional Review Board at the American University of Beirut given that it is not considered human subject research. Access to a de-identified subset of the ONL registration database was obtained from the ONL. The subset of data did not contain any personal identifiers. Data analysis was carried out in a secured setting at the premises of ONL using password-protected computers. Access to the collected data was restricted to the research team and for research purposes only.

### Data preparation

A secondary quantitative data analysis was conducted on a de-identified subset of the most updated version of the ONL’s registration database. The original registration records of the database included personal information about the nurse such as age, marital status, current address, as well as information about their professional background, including: job title, employment status, sector of employment, dates of employment, the dates of job change (if applicable), and the location or country of the previous and current employment. According to ONL, a total of 5,518 records were outdated and were consequently updated by the ONL while other records were already up-to-date and needed no further update or were categorized as “unknown” following failure to reach nurses after five call attempts. Data of all the ONL nurses’ registration records were subsequently analyzed.

### Data analysis

After receiving the updated nursing registration records, the research team analyzed a six-year de-identified subset of registration data (2009–2014). Data analysis was carried out using the SPSS version 16.0. Descriptive analysis was conducted where frequencies, percentages and percent changes were reported.

For analysis purposes, nurses’ sector of employment was divided into three main categories, each entailing different sub-sectors. Similarly, the current employment status of the nurses was divided into three main categories. The categorization of these two variables was as follow:

Employment Sector (3 categories):

1. The Hospital Sector: Includes all hospitals and Long Term Care facilities.
2. The Community Sector: Includes Primary Health Care and Home care institutions.
3. The ‘Other’ Sector: Includes the following sub-sectors:
  - “Technical and university education” sub-sectors, includes nurses in teaching positions;
  - “School and nursery nurses”;
  - “Enterprises”, includes nurses working in pharmaceutical or medical devices companies;
  - “Insurance companies”, includes nurses reporting working in insurance companies;
  - “Other”, nurses whom work setting does not fall into any of the aforementioned categories.

Current Employment Status (3 categories):

1. Active: Includes all the nurses who are registered with the ONL and who are actively working in the nursing field in Lebanon (total in 2014 = 9,115).
2. Eligible: Includes 1) nurses registered with the ONL but not working within the nursing labor market in Lebanon, such as those working in pharmaceutical companies or insurance companies; or 2) nurses who are living and/or working abroad, 3) or nurses who are unemployed (Total in 2014 = 2,577).
3. Unknown: Includes nurses originally registered with ONL but with unknown contact and employment information. Consequently their records couldn’t be verified and updated during the cleaning and updating process (Total in 2014 = 817).

## Results

**Fig 1:**
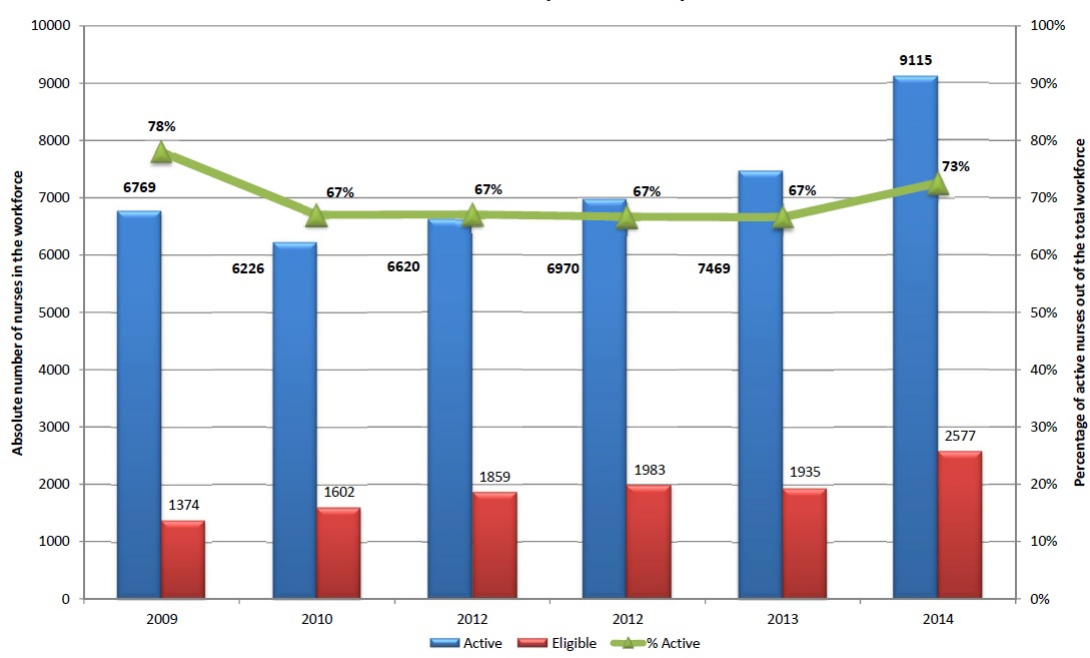
The absolute and relative distribution of active and eligible nurses in the ONL registration database (2009–2014).

Analysis of the updated ONL database showed a large increase in the size of the nursing workforce over the past six years. Between 2009 and 2014, a 35% and 86% increase in the number of active and eligible nurses were observed; respectively (Fig 1). However, the proportion of the active nurses out of the total nurses in the ONL database decreased by 5.4% throughout the period of analysis; from 78% in 2009 to 72.6% in 2014 (Fig 1).

**Fig 2:**
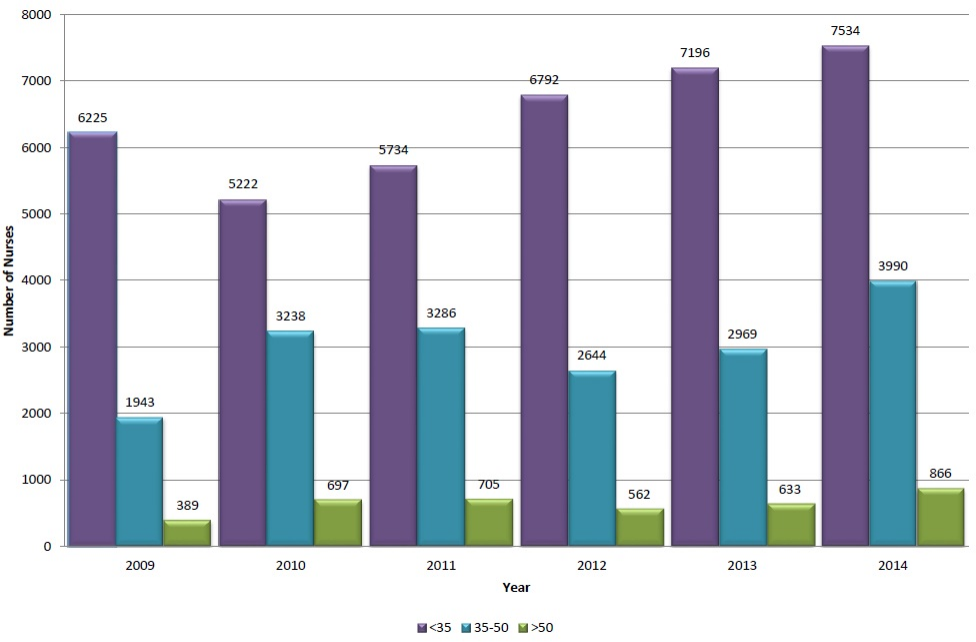
Proportional distribution of nurses registered with the ONL by age (2009–2014).

In regards to age, data analysis revealed that the nursing workforce in Lebanon is generally young with the majority of active nurses aged less than 35 years old (Fig 2). In fact only 32% and 7% of nurses in Lebanon were in the 36–50 and more than 50 age groups; respectively. In addition, the analysis of aging trends between 2009 and 2014 revealed that the proportion of nurses aged 35 years or less has actually decreased by 11.9% from 72.7% (6225 out of 8577 nurses) in 2009 to 60.8% (7534 out of 12390 nurses) in 2014. Within the same time frame, the proportion of nurses in the 35–50 grew by 9.5% from 22.7% (1943 out of 8577 nurses) in 2009 to 32.2% (3990 out of 12390 nurses) in 2014. Furthermore, the proportion of nurses in and ≥ 50 age groups grew by 2.5%, from accounting for 4.5% (389 out of 8577 nurses) of the nursing workforce in 2009 to accounting to 7.0% (866 out of 12390 nurses) of the workforce in 2014.

In regards to the gender distribution, analysis showed that the proportion of female nurses in the ONL database has remained steady at 80% throughout the period of analysis, with males constituting 20% of the Lebanese nursing workforce. In terms of distribution of nurses by type, analysis reveals that the proportion has remained stable throughout the period of analysis with four of every five nurses registered as BSN or equivalent (TS holders). Note that the period of analysis witnessed a 48.7% and 30.5% in the numbers of BSNs and BTs; respectively.

In regards to the sector of employment, a noteworthy increase in the workforce was observed in all employment sectors (Table 1). A breakdown by sector reveals that the “Hospital” sector witnessed a 38.5% growth in active nurses between 2009 and 2014, while the number of nurses working in the “Community” and “Other” increased by 54.6% and 131.5%; respectively. Even though the “Other” sector encompassed the highest proportional growth between 2009 and 2014, the “Hospital” sector remains the principle employer of the vast majority of nurses, with ONL records indicating that 86.6% of nurses in Lebanon working in the “Hospital” sector in 2014.

**Table 1:** Distribution of nurses working in Lebanon by sector and subsector of employment (2009–2014).

Sector	Sub-Sector	2009	2010	2011	2012	2013	2014	# and % Increase In Sector/ Sub-Sector Between 2009–2014
**Hospital Sector**	**Hospital**	6300	6014	6401	6724	7197	8745	(+2445) 38.8%
	**Long-Term Care**	92	102	77	82	89	108	(+16) 17.4%
	**Total**	6392	6116	6478	6806	7278	8853	**+2461 (+38.5%)**
**Community Sector**	**Primary Health Care**	304	262	313	352	364	453	(+149) 49.0%
	**Home Care**	2	12	10	10	9	20	(+18) 900%
	**Total**	306	274	323	362	373	473	**+167 (+54.6%)**
**Other Sector**	**School and Nursery Nurse**	71	77	84	85	88	157	(+86) 121%
	**Enterprises**	N/A	109	182	174	139	244	(+135) 124%^a^
	**Insurance**	23	28	29	33	40	114	(+91) 396%
	**Technical Education**	115	107	116	116	123	166	(+51) 44.3%
	**University Education**	105	115	124	137	136	156	(+51) 48.6%
	**Other**	26	12	12	15	15	59	(+33) 127%
	**Total**	340	448	547	560	541	896	**+447 (+131.5%)**

^a^ # and % Increase In Sector/ Sub-Sector between 2010–2014

The analysis of the distribution of nurses in the employment sub-sectors, portrayed an increase in the number of employed nurses in all sub-sectors. However, the highest increase was indeed recorded in the hospital sub-sector which added 2461 nurses in the period of analysis. The “Primary Health Care” sub-sector incorporates the majority of the workforce in the Community sector. The sub-sector has also witnessed a 49% growth in its nurses throughout the period of analysis. However, the absolute number of nurses working in this sector remains relatively small (only 473 nurses in 2014 equivalent to 4.6% of the active nursing workforce in Lebanon). Despite a high proportional increases recorded in the “Other” sector, the number of nurses in its various subsectors remains small. Note that the “Other” sector included a total of 896 nurses in 2014, equivalent to 8.8% of the total active nursing workforce in Lebanon.

Last but not least, the number of nurses registered with the ONL and reporting working abroad increased in both absolute and proportional terms, increasing from 245 nurses in 2010 (the first year nurses abroad were tracked) to 669 nurses in 2014; this is indeed a remarkable increase equivalent to173% (424 nurses) throughout the period of analysis.

## Discussion

This study is the first since the inception of ONL to analyze the Order of Nurses in Lebanon registration database. It presents a unique regional attempt to capitalize on the power of a national nursing association to generate essential evidence guiding the planning, organization, management, and governance of the nursing workforce.

This study revealed that the majority of nurses in Lebanon are relatively young belonging to the below 35 years age group, this is in contrast with other countries reporting an alarming ageing of the nursing workforce [9, 21]. On a positive note, this reflects a young and vibrant workforce with potentially many decades of service prior to retirement. However, the findings cast concerns on the underlying causes for this skewed age distribution, which may be indicative of the attrition of nurses in older age groups from the labor market. Potential reasons include a combination of push factors including poor salary and benefits coupled with a sub-optimal organization of the profession and a poor quality of work environment in a large proportion of institutions. Such push factors are met with lucrative pull factors in the rich Gulf Cooperative Council Countries with the offering of higher salaries and benefits and better access to career growth and professional development opportunities (15–19). In addition, recent research evidence reveals that younger nurses are often subjected to different types of verbal abuse and physical violence in their work environment which could contribute to them exiting the profession and possibly changing their career path [22]. Consequently, a high proportion of nurses decide to either leave the profession or leave the country explaining the higher proportion of nurses in the less than 35 years age group. This raises concerns on the longevity of nurses in the profession and the sustainability of the nursing workforce, underlining the need to further investigate the general reasons for their attrition, with a particular focus on the context-specific factors. Additionally, the small proportion of nurses in the older groups considerably restricts the access of younger nurses to proper mentorship and support [23, 24].

Furthermore, a steady growth in the overall workforce has been observed throughout the six-year period. The growth in the nursing workforce can be attributed to a better detection and follow-up of nurses working in the different sub-sectors, as well as the increased supply of nurses in Lebanon and the strong role of ONL advocacy for nurses. The Order’s regulations and mandatory registration system have helped a lot in unveiling the exact number of active nurses. In addition, better orientation in schools have supported with improvement in the image of the profession and the attraction of students into the profession through targeted student recruitment campaigns conducted by ONL since 2009. Similar experiences have been reported in other countries, such as the United States whereby educational reforms coupled with national image improvement and recruitment campaigns led to a remarkable growth in the applicants and enrollments in both graduate and undergraduate nursing programs [25, 26].

Analysis reveals a 35% growth in the number of active nurses in Lebanon between 2009 and 2014. However, this growth was accompanied with larger growth in the proportion of eligible nurses (86%) who accounted for 22–33% of the registered ONL workforce in any particular year. A growth in the proportion of eligible nurses has also been reported in other countries [27]. While the underlying causes for the presence of such a proportion of eligible nurses need to be systematically investigated, eligible nurses present a great opportunity to swiftly replenish the labor market with readily available nurses to cover existing vacancies and address current shortages [27]. ONL vision (2012–2015) positioned and profiled nurses in advanced roles, publicized their success stories and showed their impact in an attempt to retain nurses in Lebanon. The ONL, healthcare organizations and the Ministry of Public Health started working collaboratively to design and implement initiatives that match eligible nurses to vacancies at healthcare organizations. In 2014, ONL devised a job matching system on its Website to support the matching of unemployed nurses and those looking for a job change with potential employers in the market. Technology may support such matching initiatives, an example of which is the European Skills, Competences, Qualifications, and Occupations (ESCO) portal which was developed in Europe in 2014 in an attempt to enhance competence- based job matching of unemployed personnel to different vacancies [28].

Nurses who had their current employment status as eligible in this study included also registered nurses who migrated and are working abroad. Despite the positive increase in the nursing workforce in Lebanon, migration and attritions remain major issues in the nursing labor market worth addressing. According to literature, economic incentives have been noted as the primary motive behind pushing nurses towards recipient countries [29, 30]. In addition, professional growth and education opportunities were also seen to encourage nurses to migrate to other countries [31, 32]. Therefore, the proper understanding of the career trajectories of nurses especially those who migrate is essential in guiding the workforce planning process and future studies that would explore the factors encouraging nurses to migrate and the forces that would bring them back must be carried out [33].

With respect to employment sectors, analysis reveals that a steady vast majority of nurses in Lebanon were working in the hospital sector, which employed 86.6% of Lebanese nurses in 2014. This is not in congruence with the global trend of decreasing the proportion of nurses employed in the hospital sector due to the shift in modes of care from hospital to the community [27, 34]. This trend can be mostly linked with better job stability, higher salaries and benefits, opportunity of career development and defined job scopes that are presented in the hospital sector as compared to the community sectors [27, 35]. The increase in nurses in the “Other” sector can be attributed to the flexible working hours and better packages that are offered in this sector as compared with both the hospitals or community sectors. The aforementioned distribution of Lebanese nurses across sectors highlights a need to enhance the job offerings of nurses in the Community sector and to improve the work conditions in this sector in order to support the health sector strategy to offer universal health coverage and strengthen primary and Community care in Lebanon.

The finding that 20% of the nurses in Lebanon are males is worth noting as this is much higher than the numbers reported in other developed and developing countries such as the United States where only 8.4% of the nursing workforce are males [7]. This finding is highly desirable because higher diversity and better representation of males in the nursing workforce is perceived to improve gender-equitable access and quality of care [7]. Potential reasons of these gender figures may be linked to cultural preferences where some male patients may prefer receiving care from male nurses rather than female nurses (e.g., male patients admitted to the urology unit), or other factors such as the promising future of the nursing profession, and the financial aids and scholarships granted by universities for students who choose to pursue nursing education in order to meet the demand of the Lebanese nursing market. Yet, generally, the nursing profession was traditionally perceived as a female oriented profession, particularly in the Arab countries which falsely imposed a stereotype on the profession. The findings in this study, highlight an opportunity to further investigate, the nursing profession in Lebanon from a gender lens in order to identify the incentivizing factors that motivate the male nurses to join the nursing profession and those that enhance the sustainability and continuity of their practice. Efforts should also be invested in encouraging more males to join the workforce and in supporting the retention of the currently practicing male nurses in order to promote and enhance gender-equitable recruitment and retention practices in the nursing field.

## Conclusion

Nurses represent a fundamental professional group among health human resources and play a leading role in healthcare provision and delivery. Different strategies of nurses’ retention remain therefore crucial to thoroughly investigate. The findings of this study call on policy and decision makers to examine the underlying causes for nurses’ attrition from the labor market in Lebanon and the initiatives needed to keep nurses working throughout their professional lifespan. There is a need to investigate the push and pull factors that are affecting nurses and the design of policies and interventions that would encourage nurses to remain active in Lebanon. Furthermore, policies and interventions that would create employment opportunities outside hospitals, especially in the Community sector, are recommended.
